# An Iterative Process for Identifying Pediatric Patients With Type 1 Diabetes: Retrospective Observational Study

**DOI:** 10.2196/18874

**Published:** 2020-09-04

**Authors:** Heather Lynne Morris, William Troy Donahoo, Brittany Bruggeman, Chelsea Zimmerman, Paul Hiers, Victor W Zhong, Desmond Schatz

**Affiliations:** 1 Department of Health Outcomes and Biomedical Informatics University of Florida Gainesville, FL United States; 2 Department of Medicine University of Florida Gainesville, FL United States; 3 Division of Nutritional Sciences Cornell University Ithaca, NY United States; 4 Department of Pediatrics University of Florida Gainesville, FL United States

**Keywords:** computable phenotype, type 1 diabetes, electronic health record, pediatric

## Abstract

**Background:**

The incidence of both type 1 diabetes (T1DM) and type 2 diabetes (T2DM) in children and youth is increasing. However, the current approach for identifying pediatric diabetes and separating by type is costly, because it requires substantial manual efforts.

**Objective:**

The purpose of this study was to develop a computable phenotype for accurately and efficiently identifying diabetes and separating T1DM from T2DM in pediatric patients.

**Methods:**

This retrospective study utilized a data set from the University of Florida Health Integrated Data Repository to identify 300 patients aged 18 or younger with T1DM, T2DM, or that were healthy based on a developed computable phenotype. Three endocrinology residents/fellows manually reviewed medical records of all probable cases to validate diabetes status and type. This refined computable phenotype was then used to identify all cases of T1DM and T2DM in the OneFlorida Clinical Research Consortium.

**Results:**

A total of 295 electronic health records were manually reviewed; of these, 128 cases were found to have T1DM, 35 T2DM, and 132 no diagnosis. The positive predictive value was 94.7%, the sensitivity was 96.9%, specificity was 95.8%, and the negative predictive value was 97.6%. Overall, the computable phenotype was found to be an accurate and sensitive method to pinpoint pediatric patients with T1DM.

**Conclusions:**

We developed a computable phenotype for identifying T1DM correctly and efficiently. The computable phenotype that was developed will enable researchers to identify a population accurately and cost-effectively. As such, this will vastly improve the ease of identifying patients for future intervention studies.

## Introduction

Diabetes is one of the most common chronic diseases seen during childhood and adolescence. The incidence and prevalence of diabetes mellitus has continued to increase worldwide for both type 1 diabetes (T1DM) and type 2 diabetes (T2DM), with the rise in T2DM due in large part to the obesity epidemic [1,2]. Uncontrolled T1DM leads to short- and long-term complications and early mortality [3-6].

The vast majority of the population data about the incidence, prevalence, and effects of diabetes in youth in the United States come from select sites, such as the *SEARCH for Diabetes in Youth Study* [7] and the *T1D Exchange* [8]. In the past, outside of highly manicured registries, the thorough and accurate identification of pediatric patients with T1DM versus T2DM could only be accomplished by manual clinical record review, which was both costly and time-consuming, requiring manual medical record reviews. Currently, through the use of algorithms derived from electronic health record data, accurate identification of patients with T1DM versus T2DM may be possible. One such algorithm using a subset of *SEARCH* cohort revealed a 89% positive predictive value (PPV) and a 97% negative predictive value using only ICD-10-CM codes [9]. However, this study was conducted within a self-contained data set overseen by Kaiser Permanente. As such, this does not give a comprehensive insight into patients seen at a variety of health settings using different electronic record systems. There is thus a need for timely real-world population-level monitoring of the incidence, prevalence, and disease course of diabetes in youth that includes the ability to separate T1DM from T2DM.

The overall purpose of this project was to develop and validate an algorithm to identify pediatric patients with T1DM in an efficient and accurate manner that would be valid in a real-world database outside of a closed medical system such as Kaiser Permanente.

## Methods

### Population

#### University of Florida Health

Patients eligible for inclusion in this study were aged 0-18 and seen at University of Florida Health (UF Health). The UF Health System is a medical network associated with the University of Florida with the only comprehensive pediatric facility in North Central Florida. The Integrated Data Repository (IDR) is a large-scale database that collects and organizes information across UF Health’s clinical and research enterprises. The IDR is a secure, clinical data warehouse that aggregates data from the university’s clinical and administrative information systems, including the electronic health record system. As of 2018, the IDR housed more than 1 billion observational facts across more than 1 million patients. For query 1, the IDR was utilized to identify 300 patients for the development of the computable phenotype. Similar to other studies, 100 individuals per cohort were selected (T1DM, T2DM, no diagnosis) with the *no diagnosis* classification being used as the reference group.

#### OneFlorida

The OneFlorida Clinical Research Consortium contains over 12 million unique patient records from as early as of 2012, including Medicaid claims records. This database is maintained and updated on a quarterly basis with information from partners across the state of Florida. The OneFlorida Data Trust’s repository of statewide health care data is regularly updated with the inclusion of new partners and data refreshes from existing partners. All data are cleaned, transformed, curated, and contained in a centralized data warehouse, allowing streamlined inquiries and uniform results based on high-quality data. At present, data on 15 million patients across 22 hospitals are included within the data set going back to 2012, of which approximately 4.3 million are pediatric patients aged 18 or younger across thousands of providers, clinics, practices, and multiple hospital systems throughout the state of Florida. A SAS code that was developed from the algorithm was used to identify eligible members. Previous work has demonstrated that the OneFlorida Data Trust demographics are similar to estimates reported by the US Census Bureau [10,11]. Five OneFlorida sites that did not have prescribing data were excluded. For queries 2 and 3, we limited our results to patients aged 0-18 seen within the OneFlorida Data Trust in the year 2018.

### Study Overview

In query 1, the initial algorithm for differentiating T1DM and T2DM was developed and validated with chart reviews using data from the UF Health system. Subsequently, this algorithm was utilized in the OneFlorida database (query 2).

#### Queries

##### Query 1: Computable Phenotype Algorithm Development Using UF Health IDR

For the development of the algorithm, we identified individuals in the UF Health System that would meet the criteria of having T1DM or T2DM, and a cohort with no diagnosis of either for comparison. A total of 300 random records were requested from the IDR with 100 of each of the following: T1DM, T2DM, and no diagnosis of either. The criteria for diagnosis of T1DM used diagnosis codes, medication dispensing, and laboratory results. Patients met the T1DM algorithm criteria if they were less than or equal to 18 years of age as of December 31, 2016, and fulfilled the following criteria: (1) inpatient/outpatient with ICD-9/10 for T1DM and insulin medication within 90 days or (2) inpatient/outpatient with ICD-9/10 for T1DM and glucose >200 mg/dL or (3) inpatient/outpatient with ICD-9/10 for T1DM and hemoglobin A1c > 6.5%.

The type 2 criteria differed slightly in that it involved ICD-9/10 for patients with T2DM under the age of 18. For each identified member within the 300 total records, we obtained data on age, sex, race, ethnicity, height, weight, BMI, diagnoses, location of services, and the admit date. In order to account for a number of conflicting diagnoses for individual patients, a diagnosis ratio was used to make a final diagnosis categorization (T1DM vs T2DM). Conflicting diagnosis codes occurred when patients were seen by multiple providers, or different settings, and received both a T1DM and T2DM in the electronic health record. In order to receive a designation of T1DM or T2DM, they had to have greater occurrences of one diagnosis. Diagnosis ratio designations were applied prior to the medical record review to allow for further investigation.

The data management for query 1 was managed in a REDCap database [12]. A data abstraction form was developed for use by the medical record reviewers to manually abstract data related to a diabetes mellitus diagnosis and treatment from the medical records. This form was utilized to collect demographic data and diabetes-related clinical information including the most recent record of height, weight, hemoglobin A1c, and if islet autoantibodies were present (and type).

##### Medical Record Review

A total of 3 pediatric endocrinology fellows (BB, CZ, and PH) evaluated the medical records to determine the *true* diagnosis. A total of 295 cases, with an overlap of 41 cases to assess interrater reliability, were reviewed. For quality assurance, 14% (40/295) of all records were manually abstracted by multiple reviewers (BB, CZ, or PH). Any discrepancies were adjudicated by a senior reviewer (WD). All reviewers were blinded to the diagnosis category patients were assigned to. Each reviewer accessed the patient electronic health records to evaluate the medical record thoroughly to make a final diagnosis. Patients were given a designation of T1DM if they fell into the range of clinical criteria including diagnosis at a younger age, a history of diabetic ketoacidosis, positive antibody status, lower insulin requirements, and lower BMI. Additional data were abstracted so the most up-to-date information for laboratory values was recorded. Reviewers entered all information into a REDCap database. Following the review, data were exported into SPSS and reviewed for interrater reliability. A total of 5 cases were evaluated in greater depth due to missingness, terminology, and a differing diagnosis. The sensitivity, PPV, negative predictive value, and specificity were calculated using the numerators and denominators from the medical record review.

#### Query 2: Computable Phenotype Algorithm using OneFlorida

Abstraction conducted in query 1 highlighted a number of false-positive diagnoses. In order to correctly categorize patients with other forms of diabetes (eg, cystic fibrosis–related diabetes, maturity-onset diabetes of youth, neonatal hyperglycemia), we separated patients with these diagnostic codes into a third cohort identified as *other diabetes*. We revised the algorithm to include patients with ICD-10 of Neonatal Diabetes Mellitus P70.2 instead of P61.0 for the Other DM categories. This resulted in a reduction of 5397 patients across all years (originally 9727), and 685 patients in the year 2018 alone (previously 1316).

#### Query 3: Computable Phenotype Algorithm Using OneFlorida Revised

In the initial run of the computable phenotype in the OneFlorida Clinical Research Consortium, there was an inconsistency in the number of cases of patients with T1DM and T2DM. More specifically, there were more cases of patients with T2DM than on average. We revised the algorithm to include additional pharmacy data to identify patients who met the algorithm criteria where patients with a diagnosis code of T2DM were also required to have a prescription of metformin.

## Results

### Computable Phenotype Algorithm Development Using UF Health IDR

In our first query of 300 medical records drawn from the UF Health IDR, 5 cases had no discerning diagnosis (conflicting diagnosis of T1DM and T2DM) based on the diagnosis ratio, and therefore, these were excluded from the study. A total of 295 records were reviewed. Table 1 shows the demographics of these patients.

After applying a diagnosis ratio between hospital encounters, there were a total of 131 patients with T1DM, 64 with T2DM, and 100 with no diagnosis of either. Of the 131 patients identified using the computable phenotype algorithm, abstractors confirmed a diagnosis of T1DM for 125 patients (true positive; Table 2), which yielded a PPV of 96.8% (Table 2). Upon validation with the medical record review, it was confirmed that 7 patients were incorrectly identified (false positive; Table 2) by the algorithm. These patients instead were found to have either no diagnosis (n=5) or T2DM (n=2). The final computable phenotype algorithm was determined to have a sensitivity of 95.3%. The T2DM algorithm had a lower PPV than T1DM (51.6%) but had a high sensitivity (94.3%) and specificity (97.5%).

**Table 1:** UF demographics.

Demographic		Overall (N=295)	No diagnosis (N=132)	T1DM^a^ (N=128)	T2DM^b^ (N=35)
Age, mean (SD)		10.7 (5.44)	7.8 (5.56)	12.3 (4.07)	15.4 (2.87)
**Gender**					
	Male, n (%)	134 (45.4)	63 (47.7)	60 (46.9)	11 (31.4)
	Female, n (%)	161 (54.6)	69 (52.3)	68 (53.1)	24 (68.6)
**Race**					
	Caucasian, n (%)	179 (60.7)	79 (59.8)	87 (68.0)	13 (37.1)
	African American, n (%)	62 (21.0)	33 (25.0)	10 (7.8)	19 (54.3)
	Hispanic, n (%)	31 (10.5)	11 (8.3)	19 (14.8)	1 (2.9)
	Asian, n (%)	2 (0.7)	2 (1.5)	0 (0)	0 (0)
	Multiple races, n (%)	15 (5.1)	4 (3.0)	9 (7.0)	2 (5.7)
	Missing, n (%)	6 (2.0)	3 (2.3)	3 (2.3)	0 (0)
**Treatment facility**					
	UF^c^ Health, n (%)	231 (78.3)	81 (61.4)	117 (91.4)	33 (94.3)
Autoantibodies presence, n (%)		67 (22.7)	2 (1.5)	65 (50.8)	0 (0)
**Ethnicity**					
	Hispanic, n (%)	38 (12.9)	13 (9.8)	23 (18.0)	2 (5.7)
Glucose level, mean (SD); range		153.86 (95.45); 7-555	89.43 (30.33); 7-284	207.59 (98.57); 58-555	161.11 (96.83); 64-432
Hemoglobin A1c, mean (SD); range		8.62 (2.31); 4.8-14.00	5.48 (0.58); 4.8-7.5	9.27 (1.80); 5.6-14	7.92 (2.90); 4.9-14.00

^a^T1DM: type 1 diabetes mellitus.

^b^T2DM: type 2 diabetes mellitus.

^c^UF: University of Florida.

**Table 2:** Results from query 1.

Query 1	Total reviewed, n	Total confirmed, n	Sensitivity, %	Specificity, %	Positive predictive value, %	Negative predictive value, %
T1DM^a^ case identified via CP^b^ algorithm^c^	131	124	96.9	95.8	94.7	97.6
T2DM^d^ case identified via CP algorithm^e^	64	33	94.3	88.1	51.6	99.1

^a^T1DM: type 1 diabetes mellitus.

^b^CP: computable phenotype.

^c^T1DM algorithm: sensitivity=124/124+4; specificity=160/160+7; PPV=124/124+7; NPV=160/160+4.

^d^T2DM: type 2 diabetes mellitus.

^e^T2DM algorithm: sensitivity=33/33+2; specificity=229/229+31; PPV=33/33+31; NPV=229/229+2.

### Computable Phenotype Algorithm Performance in OneFlorida

In the second query, the performance of the algorithm was tested in the OneFlorida Data Trust. Although the validity of using only ICD codes for the determination of diabetes type in youth has been demonstrated in the large integrated health system of Kaiser Permanente Southern California [9], and while our algorithm was based largely on ICD codes and did very well in the UF Health IDR, when this was run in the OneFlorida Data Trust, there were issues with appropriate categorization as described in the “Methods” section. As these numbers were not consistent with what we know about the epidemiology and biology of T1DM versus T2DM in youth [13], we undertook a revision of the algorithm.

The revised algorithm included additional pharmacy data to identify patients who met the algorithm criteria. In the revision, patients with a diagnosis code of T2DM were also required to have a prescription of metformin. The results from the final algorithm are presented in Table 3. The majority of patients identified by the algorithm had a diagnosis of T1DM (n=4246) followed by other DM (n=660) and T2DM (n=550). Patients with T1DM had an even distribution of male and female, were predominantly White (2153/4246, 50.71%), between 11 and 15 years of age (1789/4246, 42.13%), and on insulin (3907/4246, 92.02%). Patients identified as having T2DM were more likely to be female (342/550, 62.1%), other race (190/550, 34.5%), Black (241/550, 43.8%), and between 16 and 18 years of age (300/550, 54.5%). Because of the already high sensitivity and specificity of the less robust initial algorithm for T1DM, we did not do additional chart reviews for the revised algorithm.

**Table 3:** Results of final algorithm in OneFlorida.

Demographic		T1DM^a^ (N=4246)		T2DM^b^ (N=550)		Other DM (N=660)	
**Sex**							
	Female, n (%)		2120 (49.93)		342 (62.18)		326 (49.39)
	Male, n (%)		2126 (50.07)		208 (37.82)		334 (50.61)
**Race**							
	White, n (%)		2153 (50.71)		117 (21.27)		195 (29.55)
	Black, n (%)		709 (16.70)		241 (43.82)		234 (35.45)
	Asian, n (%)		23 (0.54)		N/A^c^		N/A^c^
	Other/unknown, n (%)		1361 (32.05)		190 (34.55)		229 (34.70)
**Age**							
	0-5 years, n (%)		253 (5.96)		N/A^c^		512 (77.58)
	6-10 years, n (%)		895 (21.08)		N/A^c^		31 (4.70)
	11-15 years, n (%)		1789 (42.13)		240 (43.64)		66 (10.00)
	16-18 years, n (%)		1309 (30.83)		300 (54.55)		51 (7.73)
Insulin, n (%)		3907 (92.02)		284 (51.64)		63 (9.55)	

^a^T1DM: type 1 diabetes mellitus.

^b^T2DM: type 2 diabetes mellitus.

^c^N/A: no available data (ie, no patients identified).

## Discussion

### Principal Findings

Overall, the computable phenotype we developed to identify pediatric patients with T1DM was effective using data within the electronic health record. The identification of patients with diabetes can be complex and conflicting diagnosis codes make it even more difficult to disentangle an accurate classification. As such, the use of additional clinical parameters to narrow the focus to a specific population refines the specificity of the algorithm. For T1DM, this includes laboratory values (A1c ≥ 6.5, glucose ≥ 200 m/g).

For the purposes of this study, we drew upon the parameters already defined by the SEARCH study which allows researchers to identify adults with T1DM. Referencing this study, we made refinements to account for variations among pediatric patients. The utility of this computable phenotype is that it enables us to identify patients with an accuracy of 97%. Identification of patients solely based on the data found within the electronic health record can be complex, thus accounting for our need of numerous queries. The idiosyncrasies of diagnosis codes and limited recordings of HbA1c for patients added complexities to the methods of identification. In our experience, diagnosis codes for patients often had contradictions. For example, a patient seen multiple times in the measurement year in various settings may have conflicting diagnosis (ie, T1DM and T2DM). To overcome this problem, we applied a diagnosis ratio to include the most prevalent diagnosis. This is an important consideration for other individuals utilizing electronic health records for identification. The identification of pediatric patients solely based on the ICD-9 or ICD-10 code only allows us to look at patients on the surface level rather than as a whole.

The findings from this study were instrumental in developing a computable phenotype to identify pediatric patients with T1DM. Through this process, a number of limitations were of note that should be considered. First, the utilization of the electronic health record presented a few obstacles that were not originally foreseen, particularly the conflicting diagnoses of patients. Inaccuracies and data entry error are plausible within large data sets and need to be accounted for. Being aware of the possibility of inaccurate diagnoses increases the importance of not relying solely on ICD-9 and ICD-10 diagnoses for identifying patients. Similarly, this impacted our proposed methodology of 100 individuals for each of the 3 cohorts (ie, T1DM, T2DM, and no diagnosis). These differences were accounted for in our calculations of predictive value, sensitivity, and specificity, but still need to be noted as a potential limiting factor. Another limitation of this paper is that the medical record review was limited to 1 health care system. While we were able to identify all pediatric patients within the OneFlorida Clinical Research Consortium with T1DM, we were unable to access individualized records within each of the contributing data centers and thus unable to conduct medical record reviews at each site. Additionally, as 5 OneFlorida sites did not have prescribing data, this limits our available data, and generalizability, from the entire state of Florida.

### Conclusions

In summary, the computable phenotype that we developed to identify pediatric patients with T1DM is both accurate (PPV=96.8%) and sensitive (95.3%). This computable phenotype will enable future researchers to not only identify a population of interest accurately, but also cost-effectively. As such, this will allow for more precise implementation of interventions to help improve both clinical and psychosocial care, and ultimately improve outcomes important to patients.

## Abbreviations

IDR: Integrated Data Repository
PPV: positive predictive value
T1DM: type 1 diabetes mellitus
T2DM: type 2 diabetes mellitus
UF Health: University of Florida Health system
